# Effect of a structured teaching programme on mothers’ knowledge and utilisation of oral rehydration solution

**DOI:** 10.4102/phcfm.v17i1.4717

**Published:** 2025-05-30

**Authors:** Stephen Nanbur, Clement K. Dongurum, Godwin Achema, Emmanuel Andy, Sopen Chunuan, Kumzhi P. Ringkat, Kenai A. Nanchak, Nanvyat Nannim

**Affiliations:** 1 Department of Nursing Sciences, College of Health Sciences, University of Jos, Jos, Nigeria; 2 Department of Geography and Planning, Faculty of Environmental Sciences, University of Jos, Jos, Nigeria; 3 Community Education Unit, Queensland Ambulance Services, Queensland, Australia; 4 Department of Maternal Newborn Nursing and Midwifery, Faculty of Nursing, Prince of Songkla University, Hat Yai, Songkhla, Thailand; 5 Department of Nursing, Plateau State College of Nursing Sciences, Vom, Jos, Nigeria; 6 Department of Zoology, Faculty of Natural Sciences, University of Jos, Jos, Nigeria

**Keywords:** childhood diarrhoea, knowledge, oral rehydration solution, structured teaching programme, utilisation

## Abstract

**Background:**

Childhood diarrhoea is a major health problem in developing countries.

**Aim:**

The aim of this study was to evaluate the impact of a structured teaching programme on mothers’ knowledge and use of oral rehydration solution in the treatment of diarrhoea in children under 5 years of age.

**Setting:**

The study was conducted at Life-changing Eudaimonia Hospital, Jos, Nigeria.

**Methods:**

A quasi-experimental research design was used, based on a pre- and post-test with one group. Seventy mothers of children under 5 years of age suffering from diarrhoea were recruited as subjects. However, two withdrew, resulting in a response rate of 97.1%. Data were collected by administering a pretest to the respondents and a post-test after a 3-h structured teaching programme on the preparation and utilisation of oral rehydration solution in the treatment of diarrhoea.

**Results:**

The *t*-test analysis revealed that the mean knowledge and utilisation of oral rehydration solution in the treatment of diarrhoea significantly increased, with paired *t*-values of 3.528 (*p* = 0.001) and 20.382 (*p* < 0.001) respectively.

**Conclusion:**

We concluded that the structured teaching programme significantly improved mothers’ knowledge and utilisation of oral rehydration solution in the management of diarrhoea in children under 5 years of age at Life-changing Eudaimonia Hospital, Jos.

**Contribution:**

Based on the findings of this study, we suggest that policy makers should develop programmes that support education campaigns on oral rehydration therapy among family caregivers, especially in rural areas with poor access to health care.

## Introduction

Worldwide, diarrhoea is the leading cause of death in children under 5 years of age.^1^ Most diarrhoea-related deaths occur in South Asia and sub-Saharan Africa, particularly in Nigeria, Ethiopia, Democratic Republic of Congo, Pakistan and India.^1,2,3^ In Nigeria, several vulnerable children die from diarrhoeal diseases. Peter and Umar,^4^ reported a prevalence rate of 20% in children under 5 years of age in Nigeria. Children in the north of Nigeria are at higher risk compared to the southern part of the country.^5,6^ In addition, recent evidence suggests that diarrhoea is still a major public health problem among children in Plateau State, Nigeria.^7^ The annual incidence of diarrhoea in children aged under 5 years in Plateau State between 2013 and 2017 ranged between 13% and 24%.^6^ The proportion of children under 5 years of age diagnosed with diarrhoeal disease at Life-changing Eudaimonia Hospital, Jos, had also increased in the previous 4 years from 23% to 26.6% based on unpublished internal data.

Diarrhoea refers to the passage of three or more loose stools within 24 h.^8,9^ It can be brought about by a range of bacterial, viral and parasitic pathogens and can spread from person to person because of unhygienic habits or tainted food or water.^10,11^ Diarrhoea can be transmitted by contaminated water and food, unwashed hands and feeding utensils such as bottles and teats.^12,13^ The cycle begins when the infectious agent multiplies in the food medium. Subsequently, humans get infected upon ingestion of the contaminated food.^11^ Risk factors include unhygienic practices such as open defecation and sourcing water from the river or well.^14,15,16^ Childhood diarrhoea can be managed by fluid replacement.^17^

Oral rehydration therapy has been the major treatment regimen for diarrhoea since its invention in 1970s.^4,18^ The treatment package is centred around fluid replacement to prevent dehydration.^19,20^ Oral rehydration solution (ORS) is made by mixing specific amounts of chloride, sodium, glucose, potassium, and alkali (bicarbonate or citrate) with portable water, as directed by the manufacturer.^21^ All forms of dehydration can be effectively managed with oral rehydration therapy, using the World Health Organization (WHO) formula.^22,23^ Because of its exceptional efficacy in managing diarrhoea, it has made a significant contribution to the decrease in paediatric diarrhoeal disease case fatality rate.^23,24,25,26^ Despite the proven efficacy of ORS, its utilisation by family caregivers in the tropics is still subnormal.^27^

Family engagement in health education is essential to improve knowledge and utilisation of ORS.^28^ As primary caregivers, mothers play a significant role in managing diarrhoea in children under five.^29^ Therefore, enhancing their understanding of ORS, and its utilisation, can significantly impact health outcomes.^22,30^

Structured teaching programmes have been shown to effectively increase knowledge and change behaviours regarding health practices.^31^ Research indicates that educational interventions tailored to the needs of mothers can lead to improved health literacy and better health-seeking behaviours. Structured educational interventions have significantly enhanced mothers’ knowledge and practices related to ORS utilisation in managing childhood diarrhoea.^32,33,34^ Furthermore, a systematic review highlighted the effectiveness of educational programmes in promoting the use of ORS among caregivers, suggesting that such interventions can lead to increased utilisation and improved health outcomes for children.^28^

The increasing burden of diarrhoeal disease suggests the need for more intensive intervention research to reduce the mortality rate from diarrhoeal disease in Plateau State. However, few descriptive surveys have been published on the knowledge and utilisation of ORS in Plateau State. To our knowledge, there is no published intervention study supporting the use of oral rehydration therapy in the treatment of diarrhoeal disease in Plateau State. Against this background, our study investigated the effectiveness of a structured teaching programme on maternal knowledge and utilisation of ORS in the treatment of diarrhoea in children under 5 years of age at Life-changing Eudaimonia Hospital, Jos, Plateau State. By evaluating the impact of this educational intervention, we were able to contribute to the evidence base that supports family engagement in paediatric healthcare to improve the management of diarrhoea in vulnerable populations.

## Research methods and design

### Research hypothesis

- There is a significant difference between the mothers’ pre-test and post-test mean knowledge scores on ORS.
- There is a significant difference between the mothers’ pre-test and post-test mean competency scores on utilisation of ORS in the treatment of diarrhoea.

### Design

This was a quasi-experimental study with a one-group pre-test and post-test design.

### Setting

The study was conducted at the Life-changing Eudaimonia Hospital. The facility is a private medical centre in Jos, Plateau State, Nigeria. It is registered with the Nigerian Corporate Affairs Commission and licenced by the Plateau State Ministry of Health for the provision of medical and maternity services at secondary healthcare level.

### Study population and sampling technique

The target population comprised mothers of children under five with diarrhoea at Life-changing Eudaimonia Hospital during the period of the study. Based on Taro Yamane’s formula for determining sample size,^35,36^ 70 mothers were selected, using the consecutive sampling method.^37^ Two respondents withdrew from the study; hence, the response rate was 97.1%.

### Intervention

The intervention was a structured teaching programme developed by the researchers using the WHO guidelines for the preparation and utilisation of ORS.^38^

### Data collection

The instruments for data collection included a self-administered questionnaire and a checklist, which were developed by the investigator. The questionnaire was designed to assess the level of knowledge about ORS. It consisted of nine items, with a score for each item, totalling nine points. The 14-item checklist was developed to measure respondents’ ability to prepare and administer ORS to children under 5 years of age with diarrhoea. A total of 14 points were allocated for the checklist (1 point for each item). Respondents who scored less than 50% were categorised as having insufficient knowledge and incompetent users of ORS. Those who scored 50% and above met the criteria for sufficient knowledge and competent users.

The instruments were validated by six research and education experts. The scale content validity index for the questionnaire and checklist were 0.83 and 0.84, respectively. Data were collected by administering a pre-test to respondents followed by a 3-h structured teaching programme on the preparation and utilisation of ORS in managing childhood diarrhoea in English with translations in Hausa for mothers who did not understand English. The post-test was conducted immediately after the intervention.

### Data analysis

The data were analysed using the Statistical Package for Social Sciences Version 26. Descriptive statistics such as: frequency table, mean and standard deviation were used to analyse the demographic data. While the paired *t*-test was computed to test the hypotheses at 0.05 level of significance.

### Ethical consideration

Ethical approval for the study was obtained from the Jos University Teaching Hospital Health Research Ethics Committee (No. JUTH/DCS/IREC/127/XXXI/2695). Informed verbal consent was also obtained from each participant. Data privacy and confidentiality were ensured by using identification codes instead of participants’ names. The data were stored on a password-protected computer belonging to the author.

## Results

In Table 1, the descriptive result of the study shows that 58.8% of the participants were married and 38.2% were single mothers. Over 44% of mothers were between the ages of 20 and 29 years and 38.2% were between the ages of 30 and 39 years. The majority (76.5%) of the mothers reported they were Christians. Participants who had tertiary education accounted for 41.2% and 38.2% completed Secondary School. More than half (51.5%) worked full-time in business, while only 7.4% and 5.9% were housewives and civil servants, respectively. Almost all (92.6%) of the participants earned less than N100 000 (< $60) monthly, while a smaller proportion (7.4%) reported earning between N100 000 and N199 000 ($60 to $120) monthly. More than four in ten (42.6%) of respondents used sachet water, while about one-quarter (32.4%) sourced water from boreholes. Only 5.9% of respondents used bottled water and river and/or stream water. As shown in Table 2, it was found that mothers’ mean knowledge of ORS increased significantly after the intervention (*t* = 3.528, *p* = 0.001). The mean score for the utilisation of ORS also increased significantly after the intervention (*t* = 20.38, *p* < 0.001).

**TABLE 1:** Socio-Demographic characteristics of mothers of children under-five.

Background characteristics (*N* = 68)	Frequency	%
**Marital status**		
Married	40	58.8
Single	26	38.2
Widow	2	2.9
**Age (years) (Mean = 31.8, s.d. = 7.7)**		
20–29	30	44.1
30–39	26	38.2
> 40	12	17.6
**Religion**		
Christianity	52	76.5
Islam	16	23.5
**Educational level**		
Primary	14	20.6
Secondary	26	38.2
Tertiary	28	41.2
**Occupation**		
Civil servant	4	5.9
Business	35	51.5
Housewife	5	7.4
Private sector	12	17.6
Student	12	17.6
**Monthly income**		
< N100,000	63	92.6
≥ N100,000–N199,000	5	7.4
**Source of drinking water**		
Bottled	4	5.9
Sachet	29	42.6
Borehole	22	32.4
Well	9	13.2
River and/or stream	4	5.9

s.d., standard deviation.

**TABLE 2:** Paired ‘*t*’ test results on the relationship between pre-test and post-test knowledge and utilisation of ORS in managing childhood diarrhoea (*N* = 68).

Tables scores	Mean	s.d.	*T*	*P* value
**Knowledge**			3.528	0.001
Pre-test	5.79	3.896	-	-
Post-test	7.63	1.359	-	-
**Utilisation**			20.382	< 0.001
Pre-test	6.50	2.634	-	-
Post-test	11.44	2.384	-	-

s.d., standard deviation; ORS, oral rehydration solution.

## Discussion

Close to half of the respondents (44.1%) were between ages 20 and 29 years (Mean, 31.8 ± 7.7). This corresponds to the findings of earlier studies conducted in Jos Nigeria,^22^ Pakistan,^39^ India^40^ and Ethiopia.^41,42^ Most of the respondents were literate as 54 (79.4%) completed at least high (secondary) school. This agrees with the findings of Kalsoom et al.,^39^ but contrasts with the findings of other studies.^32,43^ Most respondents were married. Wubetu et al.,^41^ also found that most mothers of children under 5 years were married. Almost all the mothers belonged to the low socioeconomic class (earning < 60 USD monthly). Similarly, Sunanda et al.,^32^ found that most mothers of children under the age of five belonged to the lower socio-economic class. In contrast, a study reported that most mothers were in the middle socio-economic class.^39^ Most women were engaged in businesses while only a few were housewives and public servants. However, other studies reported that most mothers were housewives^39,44^; while others did not mention the occupation of participants.^22,45^

Furthermore, the findings of this study showed that the mean knowledge score of mothers improved significantly from 5.79 to 7.63 after the structured teaching programme. Similarly, there was a significant improvement of the mean score of ORS utilisation after the intervention. This implies that the structured teaching programme was effective in enhancing mothers’ knowledge and utilisation of ORS in managing childhood diarrhoea. This finding corresponds with the reports of other studies in Nigeria,^29,46^ India^32,40^ and Egypt.^44^

The link between structured teaching programme and improvement in knowledge, which leads to increased utilisation is often supported by statistical analyses.^32,47^ Paired *t*-tests and Chi-square tests are commonly employed to assess relationship between knowledge and utilisation of ORS. These analyses consistently show that educational interventions lead to significant increase in knowledge and utilisation of ORS.^29,48,49^ By increasing knowledge and understanding of ORS, structured teaching programme contribute to better management of diarrhoea and reduce the incidence of dehydration in children.^48,50,51^ Although most studies support the connection between ORS preparation expertise and its application in the treatment of diarrhoea,^22,52^ it is important to note that a recent study reported an insignificant association between knowledge and utilisation of ORS in managing childhood diarrhoea.^53^

## Nursing implication

Nurses and midwives can utilise the structured teaching programme to improve mothers’ knowledge and awareness regarding the prevention and home management of diarrhoea in under-five children. This can lead to increased utilisation of ORS by mothers in managing childhood diarrhoea, which is crucial because oral rehydration therapy has been recognised as a cost-effective and life-saving intervention in diarrhoea management.

### Strengths and limitations of the study

One group was used in the study, which made data collection simple, easy, precise and inexpensive. However, the evidence for a cause–effect relationship is weak as the study does not have a control group for comparison. Future researchers could therefore include a control group in their studies to rule out the influence of extraneous variables on the outcome of the intervention.

## Conclusion

The educational intervention significantly improved the knowledge and utilisation of ORS in managing childhood diarrhoea among mothers of children under-five years at the Life-changing Eudaimonia Hospital, Jos. This study stands out as a pioneer intervention aimed at enhancing the knowledge and practice of oral rehydration therapy in managing childhood diarrhoea in Plateau state, Nigeria. Stakeholders can leverage on the result of this study to develop policies that support structured teaching programme on oral rehydration therapy among family caregivers most especially in the rural areas where there is poor access to healthcare.
